# Expression of Concern: MiRSNP rs188493331: A Key Player in Genetic Control of MicroRNA‐Induced Pathway Activation in Hypertrophic Scars and Keloids

**DOI:** 10.1111/srt.70173

**Published:** 2025-05-05

M. Chen, Y. Pan, Z. Chen, F. Qi, J. Gu, Y. Qiu, A. He, and J. Liu, “miRSNP rs188493331: A Key Player in Genetic Control of MicroRNA‐Induced Pathway Activation in Hypertrophic Scars and Keloids,” *Skin Research and Technology* 30, no. 5 (2024): e13686, https://doi.org/10.1111/srt.13686.

This Expression of Concern is for the above article, published online on 29 April 2024 in Wiley Online Library (wileyonlinelibrary.com), and has been issued by agreement between *Skin Research and Technology*; and John Wiley & Sons Ltd. Post‐publication, it was observed that the data supporting the differential gene expression analysis—which the authors state to have conducted to compare hypertrophic scar (HS) and keloid tissues with normal skin—were not included in the article. Upon inquiry, the authors clarified that they manually screened results from previously published datasets, utilizing a single‐cell RNA sequencing dataset for keloid tissues and a separate single‐cell RNA sequencing dataset for normal skin. However, no dataset corresponding to HS tissues was reported. Furthermore, as the analysis was conducted on independent datasets without proper normalization and adjustment for direct comparison, the reliability of the results obtained through manual screening may be affected. The authors acknowledged their limited familiarity with analytical tools that could have facilitated a more robust integration of data from different sources. Consequently, the scientific validity and relevance of the identified hub genes—selected through literature and database searches—would benefit from further rigorous validation. Therefore, the journal has decided to issue an Expression of Concern to inform and alert the readers about these issues.

